# Evaluation of vaccination herd immunity effects for anogenital warts in a low coverage setting with human papillomavirus vaccine—an interrupted time series analysis from 2005 to 2010 using health insurance data

**DOI:** 10.1186/s12879-017-2663-7

**Published:** 2017-08-14

**Authors:** Kathrin Thöne, Johannes Horn, Rafael Mikolajczyk

**Affiliations:** 1 Leibniz-Institute for Prevention Research and Epidemiology – BIPS, Achterstr. 30, 28359 Bremen, Germany; 2 Hubertus Wald Tumor Center, University Cancer Center Hamburg (UCCH)/ University Medical Center Hamburg-Eppendorf (UKE), Martinistr. 52, 20246 Hamburg, Germany; 3 Helmholtz Centre for Infection Research, Inhoffenstr. 7, 38124 Braunschweig, Germany; 4 Hannover Medical School, Carl-Neuberg-Str. 1, 30625 Hannover, Germany; 5 Institute for Medical Epidemiology, Biometrics, and Informatics, Medical Faculty of the Martin-Luther University Halle-Wittenberg, 06112 Halle (Saale), Germany

**Keywords:** Human papillomavirus (HPV) vaccine, Anogenital warts (AGWs), Vaccine coverage, Herd immunity, Indirect vaccination effect

## Abstract

**Background:**

Shortly after the human papillomavirus (HPV) vaccine recommendation and hence the reimbursement of vaccination costs for the respective age groups in Germany in 2007, changes in the incidence of anogenital warts (AGWs) were observed, but it was not clear at what level the incidence would stabilize and to what extent herd immunity would be present. Given the relatively low HPV vaccination coverage in Germany, we aimed to assess potential vaccination herd immunity effects in the German setting.

**Methods:**

A retrospective open cohort study with data from more than nine million statutory health insurance members from 2005 to 2010 was conducted. AGW cases were identified using ICD-10-codes. The incidence of AGWs was estimated by age, sex, and calendar quarter. Age and sex specific incidence rate ratios were estimated comparing the years 2009–2010 (post-vaccination period) with 2005–2007 (pre-vaccination period).

**Results:**

Incidence rate ratio of AGWs for the post-vaccination period compared to the pre-vaccination period showed a u-shaped decrease among the 14- to 24-year-old females and males which corresponds well with the reported HPV vaccination uptake in 2008. A maximum reduction of up to 60% was observed for the 16- to 20-year-old females and slightly less pronounced (up to 50%) for the 16- and 18-year-old males. Age groups outside of the range 14–24 years demonstrated no decrease. The decrease of incidence occurred in both sexes early after the vaccine recommendation and stabilized at lower levels in 2009–2010.

**Conclusions:**

A relative reduction of up to 50% among males of approximately similar age groups as that of females receiving the HPV vaccination suggests herd protection resulting from assortative mixing by age. The early decrease among males can be reduced over time due to partner change.

**Electronic supplementary material:**

The online version of this article (doi:10.1186/s12879-017-2663-7) contains supplementary material, which is available to authorized users.

## Background

Infections with the human papillomavirus (HPV) are the most frequent sexually transmitted viral infections worldwide affecting both men and women [1]. HPV types 6 and 11 account for over 90% of anogenital warts (AGWs) [2]; HPV types 16 and 18 are responsible for 70% of all cervical cancers [3].

In 2006, a quadrivalent vaccine against HPV 6, 11, 16, and 18 was approved by the European Medicines Agency (EMA) for the prevention of cervical cancer. In Germany, both, the bivalent as well as the quadrivalent HPV vaccination have been recommended for girls between 12 and 17 years of age by the German Standing Vaccination Committee (STIKO) since March 2007. In Germany, both, the bivalent as well as the quadrivalent HPV vaccination have been recommended for girls between 12 and 17 years of age by the German Standing Vaccination Committee (STIKO) since March 2007. The decision, which of the two vaccines to use, is jointly made by the physicians and their patients. In reality, the German market is strongly dominated by the quadrivalent HPV vaccine (90% of the market share) [4].

Clinical trials have shown HPV vaccine efficacy of 90–100% for preventing persistent and incident HPV infections [5] and AGWs (quadrivalent vaccine only) [6]. The latter develop rapidly after HPV infection [7, 8], which enables to monitor trends in AGW incidence rates as an indicator of the HPV vaccine impact, while the incidence estimation indicating a protection against cervical cancer requires longer follow-up times [7].

Recent studies in Australia [7, 9–11], Europe [2, 8, 12–15], and the United States [16] reported a AGW incidence reduction of up to 90% in the vaccine-recommended age group, but most of them were conducted in countries with a high vaccine coverage of 70 to 90% [2, 7, 9–11, 14, 15]. Some studies also reported decreasing incidence in older age groups of females [7, 9–12, 15, 16] as well as in males [7, 10, 11, 14–16], suggesting effects of herd immunity dependent on vaccine coverage.

Since HPV vaccine introduction in Germany, controversial discussions on effectiveness and safety may have led to uncertainty among young women, their parents and maybe also among physicians [17] resulting in a limited HPV vaccine uptake [18]. It has been reported, that vaccination recommendations by physicians have a great influence on the pros and cons of the decision making process of their patients [19]. Furthermore, the German health care system includes only recommendation of HPV vaccination and the individual decision is made by the physicians and the patients. While HPV vaccination is covered by health insurance companies, an immunization program conducted by e.g. school-based health centers, primary care centers or community centers which has led to higher vaccination rates in other countries does not exist in Germany. One year after the STIKO recommendation, HPV vaccine uptake in Germany with at least one vaccine dose was about 32.2% in 12- to 17-year-old females [20]. In 2012, a similarly low vaccine uptake was reported ranging from 6.1% in 12-year-old females to 47.6% in 16-year-old females [21]. These numbers are low compared to other countries with introduced HPV vaccinations. While changes in incidences of AGWs shortly after the HPV vaccine introduction were previously studied in Germany [13], effects on herd immunity were not yet investigated, and it is not clear to what extent such effects are present in a German low coverage setting. The purpose of this study is to investigate changes in AGW incidence and potential herd immunity effects of HPV vaccination over the years 2005 to 2010 in Germany.

## Methods

### Data source

This study was conducted using data from the German Pharmacoepidemiological Research Database (GePaRD) which has been described elsewhere [22–24]. Briefly, GePaRD consists of records of four statutory health insurance providers (SHIs), including data of 17 million insurants of all ages (nearly one fourth of the population in Germany) and covering all geographical regions of Germany. In the current analysis, claims data of one of the four SHIs was used, including more than nine million insurants, covering about 8.5% of the German population. The membership in an SHI is compulsory for employees with an annual income below a predefined threshold (approximately 44,000€ in 2009). The majority of the German population is insured in SHIs (approximately 85%). There may be some overrepresentation of patients with middle to higher socio-economic status than in the average population in our data of one big countrywide SHI which is more likely to insure these patients. The database contains demographic data, in- and outpatient diagnoses recorded according to the International Classification of Diseases (ICD-10-GM), in- and outpatient diagnostic and therapeutic procedures, and outpatient drug prescriptions. Outpatient diagnoses can only be allocated to a quarter of a year and not to an exact date. Utilization of health insurance data for scientific research is regulated by the Code of Social Law in Germany. The use of the data for this study was approved by the contributing SHI and the regulatory authority. Informed consent was not required by law.

### Study design

To estimate the incidence rates of AGWs, an open cohort design was applied. Cohort entry was on January 1, 2005 or on the first day after a patient had a continuous insurance time of 12 months without an AGW diagnosis during this period and had at least his/her 11th birthday in the year of cohort entry. For the calculation of person-time under risk, cohort exit was defined as the end of the study period (i.e. December 31st, 2010), December 31st of the year in which the patient had his/her 80th birthday, interruption of insurance time for more than 3 days, end of insurance (incl. Death), or the date of an AGW diagnosis. Cohort re-entry was not possible. Age at diagnosis was calculated by subtracting the year of birth from the year of the incident diagnosis - 1: e.g. for a person born in 1979 and diagnosed with AGW in 2008: 2008–1979 – 1 = 28. According to this definition, in 2008 this person was at least 28 years old and had his/her 29th birthday.

### Case definition

Incident AGW diagnoses (ICD-10-GM A63.0) were identified via both inpatient and outpatient diagnoses, that were not proceeded by an earlier AGW diagnosis in previous 12 months. For inpatient data, all admission and discharge diagnoses were considered. For outpatient data all diagnoses coded as ‘certain’ and ‘suspected’ were taken into account.

### Statistical analysis

The incidence of AGWs for each calendar quarter was estimated for the years 2005 to 2010 by dividing the number of incident cases in each stratum by the total person time of the respective stratum, expressed as per 100,000 person-years (PY). A narrower time interval was not possible, as outpatient diagnoses representing the majority of AGW diagnoses were only reported quarterly. 95% confidence intervals (CIs) for incidence rates were calculated by the substitution method [25]. Based on a previous analysis, in which we studied changes in AGW incidence immediately following the recommendation of vaccination [13], we defined two time intervals: 1) before the recommendation of vaccination (1st quarter 2005 to 2nd quarter 2007) and 2) after uptake of vaccination was established (1st quarter 2009 to 4th quarter 2010). For the two time intervals, we estimated average incidence of AGW in a given age group and calculated the relative incidence ratio for the period after establishing the vaccination compared to the pre-vaccination period, and its 95% CI. For testing and calculation of confidence intervals, we used Poisson regression model. This analysis was restricted to males and females aged 11 to 30 years. All statistical analyses were conducted using SAS statistical software, version 9.3.

## Results

About 5 million insurance members aged 11 to 79 years were included in the cohort for each study year. Overall, 49,214 incident AGW cases were identified, ranging from 7288 in 2005 to 9352 in 2010 (Table 1). Overall incidence rates were approximately stable throughout the study years except in 2009 where overall incidences were slightly lower.

**Table 1:** Crude incidence rates (IRs) of anogenital warts per 100,000 person-years (PY) for 10- to 79-year-olds by sex from 2005 to 2010

Year	Sex	AGW cases	PY	IRs	CI (95%)
2005	Total	7288	43.7	166.7	162.9–170.6
2006		7972	47.8	166.7	163.0–170.4
2007		8068	47.6	169.6	195.9–173.3
2008		8644	51.2	168.8	165.3–172.4
2009		7890	50.9	155.0	151.6–158.5
2010		9352	56.2	166.5	163.1–169.9
2005	Male	3118	23.3	133.6	128.9–138.4
2006		3460	25.2	137.3	132.7–141.9
2007		3540	25.1	141.1	136.5–145.8
2008		3.826	26.6	144.0	139.5–148.6
2009		3605	26.5	136.2	131.8–140.7
2010		4510	29.4	153.3	148.8–157.8
2005	Female	4170	20.4	204.6	198.5–211.0
2006		4512	22.6	199.4	193.6–205.3
2007		4528	22.5	201.4	195.6–207.3
2008		4818	24.6	195.6	190.1–201.2
2009		4285	24.4	175.4	170.1–180.7
2010		4842	26.8	181.0	175.9–186.1

*Abbreviations*: *AGW* anogenital warts, *PY* person-years, *IRs* incidence rates, *CI (95%)* 95% confidence intervals

**Fig. 1:**
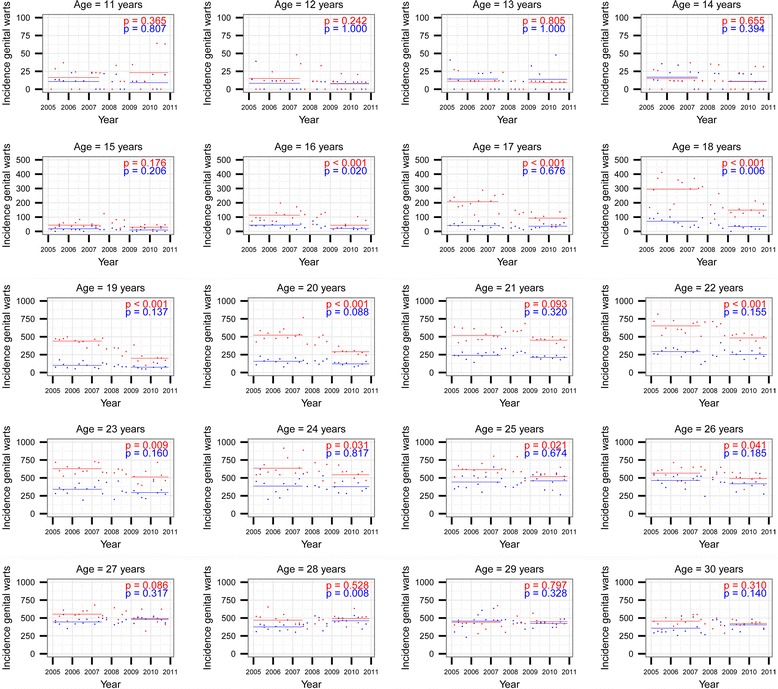
Changes in the incidence rates per 100,000 person-years (PY) of AGWs among 11- to 30-year-olds by one-year age-groups and sex (*solid lines* indicate estimated incidence in two time intervals: up to 2nd quarter of 2007 and after 1st quarter of 2009, *p*-values indicate Wald-test from Poisson regression for the indicator variable coding the two time intervals (upper (*red*) for females, lower (*blue*) for males)

Incidence and its changes varied strongly by age (Additional file 1: Table S1), making analyses stratified into 1-year age groups necessary (Fig. 1). In all age-groups, incidence was approximately stable between the 1st quarter of 2005 and the 2nd quarter of 2007. With an exception of the 21-year-old females where a statistically non-significant decrease was found, **t**he incidence decreased between the 2nd quarter of 2007 and the 4th quarter of 2008 among 16- to 26-year-old females and 16- and 18-year-old males and stabilized at a lower level afterwards. At the same time, in most other age groups, the incidence was approximately stable or increased slightly over the studied period.

**Fig. 2:**
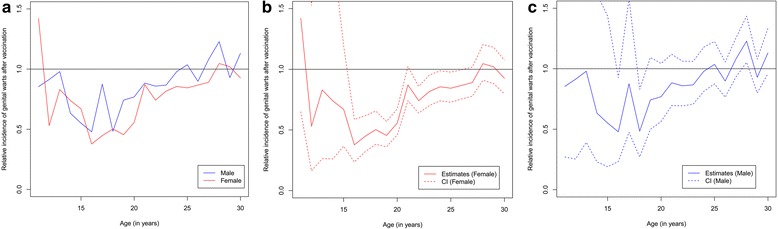
Relative incidence of AGWs by sex and age **a** overall, **b** females, **c** males (incidence 2005–2007* compared to incidence 2009–2010). (*Dotted lines* indicate 95% confidence interval). *up to 2nd quarter of 2007

When comparing the post-vaccination period with the pre-vaccination period, the relative incidence ratio indicated a u-shaped reduction of AGWs after the recommendation of vaccination in the group of 14- to 24-year-olds (Fig. 2a). The reduction was strongest for 16- to 20-year-old females (up to 60% reduction) and slightly less pronounced for 16- and 18-year-old males (up to 50% reduction (Fig. 2b and c)). In females 21 to 26 years of age a reduction of 10–20% was seen which was also found for males but estimates did not achieve significance. The reduction in the age groups 12- to 15-year-olds was about 20–30% but confidence intervals were wide.

## Discussion

The present study analyzed changes in the incidence of AGWs following the HPV vaccine recommendation in Germany and particularly the effects of herd protection among males in a low vaccine coverage setting. We found a u-shaped decrease among the 14- to 24-year-olds with a maximum reduction up to 60% observed for the 16- to 20-year-old females and a similar, but slightly less pronounced reduction for 16- and 18-year-old males.

In our analysis, the decrease of incidence occurred early after the vaccine recommendation in 2007 and stabilized after reaching a lower level in the first quarter of 2009. This reflects the finding that those who are going to be vaccinated do so in a relatively short time window. The decrease in incidence in this respective group reflects the increasing proportion of those getting vaccinated. This appears also plausible, e.g., for females aged 16, 17, or 18 years, as they had only one to 2 years in the recommended age range of vaccination and are the age groups with the most pronounced reduction in our analysis.

The u-shaped incidence reduction within the age groups of 14 to 24 years can be explained by the fact that HPV vaccination coverage increases with age within the recommended age interval from 12 to 17 years [20]. In addition, the study population originated from an SHI which reimburses the HPV vaccination in females up to the age of 26 years, explaining the reduction of incidence up to that age. Still, the uptake of vaccination closer to the upper end of this age interval is lower explaining why the effect diminishes. In the first year after recommendation, HPV vaccine uptake was about 30% for 12- to 17-year-olds, 12% for 18- to 26-year-olds, and only about 2% in 26-year-olds) [20]. One reason for low uptake or coverage rates in the older age groups (18 to 26) might be that neither the vaccination was officially recommended for these age groups nor was it stated in the vaccination schedule. Therefore, women might not have been aware that the reimbursement is possible. The maximum reduction of 60% is broadly consistent with the observed vaccination uptake based on administrative data [20, 21] or with the reported vaccination coverage based on data from several surveys [26–28], or from private insurance companies [18] in Germany. The most prominent decrease of incidence among females 16 to 20 years of age corresponds to the highest uptake in 14- to 17-year-old females of about 37% in 2008 [20]. Also the reduction in younger age groups (13 to 15 years of age) of 20 - 30% as well as in older age groups (18- to 26-year-olds) corresponds well with the reported HPV vaccination uptake in 2008 [20].

Despite the fact that the recommendation for HPV vaccination only includes females in Germany, we observed parallel effects among males of nearly the same age. Here, the most pronounced decrease was seen in 16- to 18-year-olds which is plausible as females have, on average, similarly aged or one to 2 years older sexual partners during adolescence. Other studies reported decreasing incidences of AGWs in males of the same age as vaccinated females, in a high vaccine coverage setting of about 80% or higher [10, 11, 14, 15, 29]. But also some studies with low age-specific vaccine coverage of about 50% or lower reported a decrease in AGW incidence or prevalence [30] for males younger than 20 [31] or 25 [16] years of age. This is in line with the incidence reduction of AGWs in our study for 16- to- 20-year-old females and less pronounced for 16- and 18-year-old males. A prior review including some of the above mentioned studies concluded that herd immunity effects were demonstrated only in populations with vaccine coverage of 50% or higher [32]. However, there might be differences with respect to homogeneity of vaccination coverage, for example in countries with an immunization program against HPV a percentage of girls of a certain age is vaccinated. In contrast, in Germany vaccination in general is recommended but voluntary and HPV vaccination can occur at any age between 12 to 17 years. In consequence, coverage is low for the full age-group of 12- to 17-year-olds, but in females aged 18 years vaccine coverage approaches 50%. Such heterogeneity might also explain why effects in males can be observed despite overall low coverage. While the relative changes were similar in both sexes, changes in the absolute incidence among males between pre-vaccination and post-vaccination period were less prominent. However, this might rather be a diagnostic bias than a true epidemiological difference, resulting from the fact, that females usually consult gynaecologists routinely about once a year, while males are not enrolled in a comparable system and are diagnosed only by actively consulting a physician or via an incidental finding. Thus, a higher fraction of the true incidence might be reported for females than for males, resulting in a higher incidence. In some of the age groups among males the incidence was so low, that relative reduction did not achieve significance.

The only slightly lower herd immunity effects in males than the direct protection effects in females of the same age suggest strongly assortative mating by age. The relatively short time period since the recommendation of vaccination most likely covers a single sexual relationship, which reportedly lasts for a median time of 26 months for males (interquartile range (IQR), 8.5–42) and 32 months (IQR, 9–49) for females in the relevant age groups [33]. HPV infectivity is high and in heterosexual serially monogamous partnerships, the protection of males by having a vaccinated female partner will decrease with change of partners, particularly as the reported median gap between different partners of 14 to 24 days is shorter than an HPV infection period [34]. Consequently, studies covering longer study periods particularly in low vaccination coverage settings would possibly observe diminishing protection among males while direct effects among females would remain constant. On the other hand, in high coverage settings, overall herd immunity effects may reduce the cumulative effects of change of female partners among males. In accordance with findings of other studies [2, 8, 9, 11–16], the incidence of AGWs among older age groups did not display a decreasing trend. Potential herd immunity effects in older age groups are based on sexual mixing with partners from vaccinated age groups. In a low coverage setting, the influence of such indirect protection might be too small to be detected.

In our study, the incidence of AGWs started to decrease among the 16- to 26-year-olds approximately 3 months after the vaccine recommendation by STIKO in Germany in March 2007. The short delay with which the effects of vaccination on AGW incidence were observed is consistent with the biology of infection. As HPV immunity is reported already after one vaccine dose [35] and AGWs develop after a medium incubation time of about 3 months [6–8], the corresponding time lag of decreasing incidence of one to two quarters of a year after the vaccine recommendation for females is plausible. A decreasing incidence in the target vaccination group soon after vaccine introduction has been also reported from several other ecologic studies conducted in Australia, Europe, and the US [2, 8, 9, 11–16].

We could not find herd immunity effects in older age groups (> 24 years of age) which could be explained by a limited sample size and less exclusive sexual mixing patterns of older age groups with much younger partners.

Limitations of our study are mainly related to the underlying administrative data used for analyses. For example, it was not possible to validate the diagnoses of AGWs, because we had to rely on what was used for reimbursement. Also, not all relevant variables were available in the desired detail. In particular, exact diagnosis dates in the outpatient sector are not available in the database, making the estimation of incidence less precise. It was not possible to determine the vaccine coverage of non-vaccinated persons per age cohort within the database as the vaccination status before the study period would have to be known, but was not available in our data. However, HPV vaccine uptake has been estimated in 2008 [20] and recently confirmed in a newer study in 2014 [36]. Both sources point towards a coverage of about 40–50%, or even higher if also single vaccine dose is considered. Due to data protection reasons, only the birth year but not the exact date of birth is contained in the database, which might lead to some non-differential misclassification of persons by age as some persons may already be 1 year older according to the definition of age. For the analysis of trends over time, the age of one patient is kept the same throughout all four quarters of the year, which leads to an average aging cohort from the 1st to the 4th quarter of a year resulting in higher incidences in the 4th quarter than in the 1st quarter of each year most prominently for 16- to 20-year-olds. Another limitation is that only patients who see a physician for AGWs could be identified in the database. AGWs do not necessarily cause discomfort or pain, and some patients might be embarrassed and not consult a physician. This would result in a possible underestimation of incidences of AGWs. Furthermore, AGWs may be coded as an unspecific disease, for example, as viral warts or as a sexually transmitted infection not otherwise specified, which would also result in an underestimation of AGW incidence. Finally, because of practical reasons related to data availability and permissions to use, we were unfortunately limited to analyze only data up to 2010. Particularly assessing longer trends would be of interest and should be conducted in the future.

Strengths of the study are the large population size which was not restricted to specific regions or settings in Germany and the almost complete lack of selection effects among participants, as a routine care situation is reflected in the data.

## Conclusions

In conclusion, soon after the vaccine recommendation in Germany in 2007, the incidence of AGWs decreased among 14- to 24-year-old females. In the same time there was a slightly less pronounced decrease among males of approximately the same age. The only slightly weaker effect among males suggests protection effects due to assortative sexual mixing with respect to age. The strength of this indirect protection effect might decrease over time due to partner change.

## Additional file

Additional file 1: Table S1. Person-Time, Cases and Incidence Estimates for 2005 to 2010 for Patients Aged 10 to 79 Years by Sex and Five Year Age Groups. (PDF 72 kb)

## Abbreviations

AGW: Anogenital wart
CI: Confidence interval
EMA: European Medicines Agency
GePaRD: German Pharmacoepidemiological Research Database
HPV: Human papillomavirus
ICD-10-GM: International Classification of Diseases, Version 10, German modification
IQR: Interquartile range
PY: Person years
SHI: Statutory Health Insurance
STIKO: German Standing Vaccination Committee

## References

[1] Gross G, Pfister H (2004). Role of human papillomavirus in penile cancer, penile intraepithelial squamous cell neoplasias and in genital warts. Med Microbiol Immunol.

[2] Baandrup L, Blomberg M, Dehlendorff C, Sand C, Andersen KK, Kjaer SK (2013). Significant decrease in the incidence of genital warts in young Danish women after implementation of a national human papillomavirus vaccination program. Sex Transm Dis.

[3] Smith J, Lindsay L, Hoots B, Keys J, Franceschi S, Winer R (2007). Human papillomavirus type distribution in invasive cervical cancer and high-grade cervical lesions: a meta-analysis update. Int J Cancer.

[4] Schwabe U, Paffrath D. Arzneiverordnungsreport 2009. Aktuelle Daten, Kosten, Trends und Kommentare. Berlin Heidelberg: Springer-Verlag. 2009.

[5] Harper DM, Franco EL, Wheeler CM, Moscicki A-B, Romanowski B, Roteli-Martins CM (2006). Sustained efficacy up to 4·5 years of a bivalent L1 virus-like particle vaccine against human papillomavirus types 16 and 18: follow-up from a randomised control trial. Lancet.

[6] Dillner J, Kjaer SK, Wheeler CM, Sigurdsson K, Iversen O-E, FUTURE I/II Study Group (2010). Four year efficacy of prophylactic human papillomavirus quadrivalent vaccine against low grade cervical, vulvar, and vaginal intraepithelial neoplasia and anogenital warts: randomised controlled trial. BMJ.

[7] Donovan B, Franklin N, Guy R, Grulich AE, Regan DG, Ali H (2011). Quadrivalent human papillomavirus vaccination and trends in genital warts in Australia: analysis of national sentinel surveillance data. Lancet Infect Dis.

[8] Blomberg M, Dehlendorff C, Munk C, Kjaer SK (2013). Strongly decreased risk of genital warts after vaccination against human Papillomavirus: Nationwide follow-up of vaccinated and unvaccinated girls in Denmark. Clin Infect Dis.

[9] Fairley CK, Hocking JS, Gurrin LC, Chen MY, Donovan B, Bradshaw CS (2009). Rapid decline in presentations of genital warts after the implementation of a national quadrivalent human papillomavirus vaccination programme for young women. Sex Transm Infect.

[10] Read TRH, Hocking JS, Chen MY, Donovan B, Bradshaw CS, Fairley CK (2011). The near disappearance of genital warts in young women 4 years after commencing a national human papillomavirus (HPV) vaccination programme. Sex Transm Infect.

[11] Ali H, Donovan B, Wand H, Read TRH, Regan DG, Grulich AE (2013). Genital warts in young Australians five years into national human papillomavirus vaccination programme: national surveillance data. BMJ.

[12] Leval A, Herweijer E, Arnheim-Dahlström L, Walum H, Frans E, Sparén P (2012). Incidence of genital warts in Sweden before and after quadrivalent human papillomavirus vaccine availability. J Infect Dis.

[13] Mikolajczyk RT, Kraut AA, Horn J, Schulze-Rath R, Garbe E (2013). Changes in incidence of anogenital warts diagnoses after the introduction of human papillomavirus vaccination in Germany-an ecologic study. Sex Transm Dis.

[14] Howell-Jones R, Soldan K, Wetten S, Mesher D, Williams T, Gill ON (2013). Declining genital warts in young women in england associated with HPV 16/18 vaccination: an ecological study. J Infect Dis.

[15] Sandø N, Kofoed K, Zachariae C, Fouchard J (2014). A reduced national incidence of anogenital warts in young danish men and women after introduction of a national quadrivalent human papillomavirus vaccination programme for young women - an ecological study. Acta Derm Venereol.

[16] Bauer HM, Wright G, Chow J (2012). Evidence of human papillomavirus vaccine effectiveness in reducing genital warts: an analysis of California public family planning administrative claims data, 2007-2010. Am J Public Health.

[17] Perkins RB, Clark JA (2012). What affects human Papillomavirus vaccination rates? A qualitative analysis of providers’ perceptions. Womens Heal Issues.

[18] Wild F. Impfung gegen humane Papillomaviren (HPV); Eine Analyse der Verordnungsdaten Privatversicherer. 2011; WIP-Diskussionspapier 3/2011.

[19] Bundeszentrale für gesundheitliche Aufklärung (BZgA). Elternbefragung zum Thema “Impfen im Kindesalter”. http://www.bzga.de/forschung/studien-untersuchungen/studien/?sid=10. Accessed 16 Feb 2017.

[20] Hense S, Hillebrand K, Horn J, Mikolajczyk R, Schulze-Rath R, Garbe E (2014). HPV vaccine uptake after introduction of the vaccine in Germany: an analysis of administrative data. Hum Vaccines Immunother.

[21] Rieck T, Feig M, Deler Y, Wichmann O (2014). Utilization of administrative data to assess the association of an adolescent health check-up with human papillomavirus vaccine uptake in Germany. Vaccine.

[22] Pigeot I, Ahrens W (2008). Establishment of a pharmacoepidemiological database in Germany: methodological potential, scientific value and practical limitations. Pharmacoepidemiol Drug Saf.

[23] Ohlmeier C, Frick J, Prütz F, Lampert T, Ziese T, Mikolajczyk R (2014). Use of routine data from statutory health insurances for federal health monitoring purposes. Bundesgesundheitsblatt Gesundheitsforschung Gesundheitsschutz.

[24] Garbe E, Suling M, Kloss S, Lindemann C, Schmid U (2011). Linkage of mother–baby pairs in the German Pharmacoepidemiological research database. Pharmacoepidemiol Drug Saf.

[25] Daly LE (1998). Confidence limits made easy: interval estimation using a substitution method. Am J Epidemiol.

[26] Deleré Y, Böhmer MM, Walter D, Wichmann O (2013). HPV vaccination coverage among women aged 18-20 years in Germany three years after recommendation of HPV vaccination for adolescent girls: results from a cross-sectional survey. Hum Vaccin Immunothe.

[27] Blödt S, Holmberg C, Müller-Nordhorn J, Rieckmann N (2012). Human Papillomavirus awareness, knowledge and vaccine acceptance: a survey among 18-25 year old male and female vocational school students in Berlin, Germany. Eur J Pub Health.

[28] Samkange-Zeeb F, Spallek L, Klug SJ, Zeeb H (2012). HPV infection awareness and self-reported HPV vaccination coverage in female adolescent students in two German cities. J Community Health.

[29] Chow EP, Read TR, Wigan R, Donovan B, Chen MY, Bradshaw CS, Fairley CK. Ongoing decline in genital warts among young heterosexuals 7 years after the Australian human papillomavirus (HPV) vaccination programme. Sex Transm Infect. 2015;91(3):214-9. doi:10.1136/sextrans-2014-051813. Epub 2014 Oct 10. 10.1136/sextrans-2014-051813 25305210

[30] Flagg EW, Schwartz R, Weinstock H (2013). Prevalence of anogenital warts among participants in private health plans in the United States, 2003-2010: potential impact of human papillomavirus vaccination. Am J Public Health.

[31] Oliphant J, Perkins N (2011). Impact of the human papillomavirus (HPV) vaccine on genital wart diagnoses at Auckland sexual health services. N Z Med J.

[32] Drolet M, Bénard É, Boily M-C, Ali H, Baandrup L, Bauer H (2015). Population-level impact and herd effects following human papillomavirus vaccination programmes: a systematic review and meta-analysis. Lancet Infect Dis.

[33] Widdice L, Ma Y, Jonte J, Farhat S, Breland D, Shiboski S (2013). Concordance and transmission of human papillomavirus within heterosexual couples observed over short intervals. J Infect Dis.

[34] Mercer CH, Aicken CRH, Tanton C, Estcourt CS, Brook MG, Keane F (2013). Serial monogamy and biologic concurrency: measurement of the gaps between sexual partners to inform targeted strategies. Am J Epidemiol.

[35] Kreimer AR, Struyf F, Del Rosario-Raymundo MR, Hildesheim A, Skinner SR, Wacholder S (2015). Efficacy of fewer than three doses of an HPV-16/18 AS04-adjuvanted vaccine: combined analysis of data from the Costa Rica vaccine and PATRICIA trials. Lancet Oncol.

[36] Robert-Koch-Institut. Impfquoten der Rotavirus-, Masern-, HPV-und Influenza- Impfung in Deutschland. Epidemiol Bull. 2017;1–16. https://www.rki.de/DE/Content/Infekt/EpidBull/Archiv/2017/Ausgaben/01_17.pdf?__blob=publicationFile. Accessed 16 Feb 2017.

