# Supplementary material for: Evaluation of vaccination herd immunity effects for anogenital warts in a low coverage setting with human papillomavirus vaccine—an interrupted time series analysis from 2005 to 2010 using health insurance data
Source: BMC Infect Dis. 2017 Aug 14;17:564. doi: 10.1186/s12879-017-2663-7 (PMC5557251; doi:10.1186/s12879-017-2663-7)
Supplement: Additional file 1: Table S1. — Person-Time, Cases and Incidence Estimates for 2005 to 2010 for Patients Aged 10 to 79 Years by Sex and Five Year Age Groups. (PDF 72 kb) [file 12879_2017_2663_MOESM1_ESM.pdf]

Table 1: Person-Time, Cases and Incidence Estimates for 2005 for Patients Aged 10 to 79 Years by Sex and Five Year Age Groups

	Cases	Person-Time (in 100,000 years)	Incidence (per 100,000 person-years)	95% Confidence Interval
Female	4170	20.38	204.64	[198.48; 210.95]
10 - 14 years	29	1.51	19.17	[12.84; 27.54]
15 - 19 years	423	1.36	311.62	[282.62; 342.78]
20 - 24 years	827	1.37	601.58	[561.28; 644.02]
25 - 29 years	723	1.46	495.19	[459.75; 532.64]
30 - 34 years	463	1.53	303.34	[276.33; 332.27]
35 - 39 years	585	2.45	239.07	[220.09; 259.25]
40 - 44 years	469	2.58	181.60	[165.54; 198.81]
45 - 49 years	288	1.98	145.80	[129.45; 163.65]
50 - 54 years	162	1.56	103.62	[88.28; 120.86]
55 - 59 years	75	1.23	60.73	[47.77; 76.12]
60 - 64 years	59	1.35	43.58	[33.17; 56.21]
65 - 69 years	44	1.09	40.33	[29.30; 54.14]
70 - 74 years	18	0.53	33.87	[20.08; 53.54]
75 - 79 years	5	0.37	13.65	[4.43; 31.84]

------------------------------------ continued on the next page ------------------------------------

	Cases	Person-Time (in 100,000 years)	Incidence (per 100,000 person-years)	95% Confidence Interval
------ continued ------				
Male	3118	23.34	133.57	[128.93; 138.35]
10 - 14 years	21	1.58	13.29	[8.22; 20.31]
15 - 19 years	133	1.44	92.35	[77.32; 109.45]
20 - 24 years	441	1.32	333.91	[303.47; 366.57]
25 - 29 years	534	1.40	381.46	[349.79; 415.23]
30 - 34 years	431	1.43	300.59	[272.87; 330.35]
35 - 39 years	478	2.48	193.01	[176.09; 211.11]
40 - 44 years	385	2.83	136.13	[122.87; 150.43]
45 - 49 years	223	2.31	96.60	[84.33; 110.14]
50 - 54 years	156	1.98	78.86	[66.97; 92.25]
55 - 59 years	99	1.62	61.09	[49.65; 74.38]
60 - 64 years	103	1.99	51.72	[42.22; 62.73]
65 - 69 years	77	1.68	45.78	[36.13; 57.22]
70 - 74 years	27	0.82	32.79	[21.61; 47.71]
75 - 79 years	10	0.46	21.80	[10.45; 40.09]

------ continued on the next page ------

	Cases	Person-Time (in 100,000 years)	Incidence (per 100,000 person-years)	95% Confidence Interval
---------- continued ----------				
Total	7288	43.72	166.70	[162.89; 170.57]
10 - 14 years	50	3.09	16.16	[12.00; 21.31]
15 - 19 years	556	2.80	198.74	[182.56; 215.97]
20 - 24 years	1268	2.70	470.43	[444.89; 497.05]
25 - 29 years	1257	2.86	439.52	[415.56; 464.51]
30 - 34 years	894	2.96	302.00	[282.53; 322.47]
35 - 39 years	1063	4.92	215.90	[203.12; 229.28]
40 - 44 years	854	5.41	157.83	[147.42; 168.78]
45 - 49 years	511	4.28	119.29	[109.17; 130.09]
50 - 54 years	318	3.54	89.79	[80.19; 100.22]
55 - 59 years	174	2.86	60.94	[52.22; 70.69]
60 - 64 years	162	3.35	48.43	[41.26; 56.48]
65 - 69 years	121	2.77	43.64	[36.21; 52.14]
70 - 74 years	45	1.35	33.22	[24.23; 44.45]
75 - 79 years	15	0.83	18.18	[10.17; 29.98]

Table 2: Person-Time, Cases and Incidence Estimates for 2006 for Patients Aged 10 to 79 Years by Sex and Five Year Age Groups

	Cases	Person-Time (in 100,000 years)	Incidence (per 100,000 person-years)	95% Confidence Interval
Female	4512	22.63	199.42	[193.64; 205.32]
10 - 14 years	31	1.68	18.47	[12.55; 26.22]
15 - 19 years	452	1.51	298.60	[271.70; 327.44]
20 - 24 years	878	1.45	604.53	[565.20; 645.87]
25 - 29 years	807	1.68	481.04	[448.42; 515.41]
30 - 34 years	437	1.61	270.87	[246.07; 297.50]
35 - 39 years	577	2.55	226.24	[208.16; 245.48]
40 - 44 years	575	2.93	196.29	[180.57; 213.01]
45 - 49 years	314	2.30	136.42	[121.74; 152.37]
50 - 54 years	168	1.78	94.20	[80.49; 109.57]
55 - 59 years	116	1.46	79.33	[65.55; 95.15]
60 - 64 years	67	1.33	50.52	[39.15; 64.16]
65 - 69 years	52	1.31	39.81	[29.73; 52.20]
70 - 74 years	29	0.63	46.06	[30.85; 66.15]
75 - 79 years	9	0.40	22.40	[10.24; 42.52]

------------------------------------ continued on the next page ------------------------------------

	Cases	Person-Time (in 100,000 years)	Incidence (per 100,000 person-years)	95% Confidence Interval
---------- continued ----------				
Male	3460	25.21	137.26	[132.73; 141.92]
10 - 14 years	25	1.76	14.21	[9.19; 20.97]
15 - 19 years	122	1.61	75.78	[62.93; 90.49]
20 - 24 years	446	1.39	320.07	[291.05; 351.20]
25 - 29 years	688	1.57	437.47	[405.38; 471.41]
30 - 34 years	456	1.46	312.52	[284.49; 342.57]
35 - 39 years	494	2.43	203.51	[185.96; 222.27]
40 - 44 years	444	3.07	144.57	[131.44; 158.67]
45 - 49 years	288	2.59	111.38	[98.89; 125.01]
50 - 54 years	147	2.15	68.25	[57.66; 80.22]
55 - 59 years	120	1.85	64.91	[53.82; 77.62]
60 - 64 years	105	1.90	55.35	[45.27; 67.00]
65 - 69 years	80	1.92	41.70	[33.07; 51.90]
70 - 74 years	33	0.98	33.76	[23.24; 47.42]
75 - 79 years	12	0.53	22.53	[11.64; 39.36]

---------- continued on the next page ----------

	Cases	Person-Time (in 100,000 years)	Incidence (per 100,000 person-years)	95% Confidence Interval
-- continued --				
Total	7972	47.83	166.66	[163.02; 170.36]
10 - 14 years	56	3.44	16.29	[12.31; 21.16]
15 - 19 years	574	3.12	183.76	[169.04; 199.43]
20 - 24 years	1324	2.85	465.24	[440.52; 490.99]
25 - 29 years	1495	3.25	459.96	[436.93; 483.88]
30 - 34 years	893	3.07	290.65	[271.90; 310.36]
35 - 39 years	1071	4.98	215.16	[202.46; 228.44]
40 - 44 years	1019	6.00	169.82	[159.55; 180.58]
45 - 49 years	602	4.89	123.17	[113.53; 133.41]
50 - 54 years	315	3.94	80.00	[71.41; 89.34]
55 - 59 years	236	3.31	71.28	[62.47; 80.98]
60 - 64 years	172	3.22	53.36	[45.69; 61.96]
65 - 69 years	132	3.22	40.94	[34.25; 48.54]
70 - 74 years	62	1.61	38.58	[29.58; 49.46]
75 - 79 years	21	0.93	22.48	[13.91; 34.36]

Table 3: Person-Time, Cases and Incidence Estimates for 2007 for Patients Aged 10 to 79 Years by Sex and Five Year Age Groups

	Cases	Person-Time (in 100,000 years)	Incidence (per 100,000 person-years)	95% Confidence Interval
Female	4528	22.48	201.38	[195.56; 207.34]
10 - 14 years	43	1.67	25.81	[18.68; 34.76]
15 - 19 years	433	1.37	315.78	[286.74; 346.97]
20 - 24 years	829	1.31	633.92	[591.50; 678.58]
25 - 29 years	861	1.72	499.58	[466.76; 534.10]
30 - 34 years	514	1.55	331.35	[303.32; 361.27]
35 - 39 years	519	2.33	222.35	[203.63; 242.33]
40 - 44 years	563	2.95	191.16	[175.69; 207.62]
45 - 49 years	350	2.44	143.63	[128.97; 159.49]
50 - 54 years	164	1.86	88.13	[75.16; 102.70]
55 - 59 years	118	1.54	76.72	[63.50; 91.88]
60 - 64 years	47	1.18	39.95	[29.36; 53.13]
65 - 69 years	60	1.43	42.05	[32.09; 54.12]
70 - 74 years	18	0.73	24.80	[14.70; 39.19]
75 - 79 years	9	0.42	21.41	[9.79; 40.65]

------------------------------------ continued on the next page ------------------------------------

	Cases	Person-Time (in 100,000 years)	Incidence (per 100,000 person-years)	95% Confidence Interval

-- continued --

	Cases	Person-Time (in 100,000 years)	Incidence (per 100,000 person-years)	95% Confidence Interval
Male	3540	25.09	141.10	[136.49; 145.83]
10 - 14 years	19	1.73	10.98	[6.61; 17.15]
15 - 19 years	109	1.47	74.35	[61.05; 89.69]
20 - 24 years	480	1.31	366.95	[334.85; 401.29]
25 - 29 years	679	1.57	432.61	[400.68; 466.41]
30 - 34 years	489	1.42	344.60	[314.73; 376.54]
35 - 39 years	458	2.19	209.57	[190.81; 229.67]
40 - 44 years	480	3.06	156.62	[142.92; 171.28]
45 - 49 years	293	2.71	108.23	[96.19; 121.36]
50 - 54 years	165	2.22	74.45	[63.52; 86.71]
55 - 59 years	142	1.94	73.03	[61.51; 86.07]
60 - 64 years	79	1.73	45.65	[36.14; 56.89]
65 - 69 years	95	2.04	46.57	[37.68; 56.93]
70 - 74 years	35	1.12	31.18	[21.72; 43.36]
75 - 79 years	17	0.58	29.09	[16.95; 46.58]

------------------------------------ continued on the next page ------------------------------------

	Cases	Person-Time (in 100,000 years)	Incidence (per 100,000 person-years)	95% Confidence Interval
--- continued ---				
Total	8068	47.57	169.59	[165.91; 173.33]
10 - 14 years	62	3.40	18.26	[14.00; 23.40]
15 - 19 years	542	2.84	191.03	[175.28; 207.81]
20 - 24 years	1309	2.62	500.42	[473.67; 528.28]
25 - 29 years	1540	3.29	467.66	[444.59; 491.62]
30 - 34 years	1003	2.97	337.68	[317.10; 359.24]
35 - 39 years	977	4.52	216.17	[202.83; 230.16]
40 - 44 years	1043	6.01	173.54	[163.17; 184.40]
45 - 49 years	643	5.14	125.00	[115.52; 135.04]
50 - 54 years	329	4.08	80.69	[72.21; 89.90]
55 - 59 years	260	3.48	74.66	[65.86; 84.31]
60 - 64 years	126	2.91	43.34	[36.10; 51.60]
65 - 69 years	155	3.47	44.71	[37.95; 52.33]
70 - 74 years	53	1.85	28.67	[21.48; 37.50]
75 - 79 years	26	1.00	25.88	[16.90; 37.92]

Table 4: Person-Time, Cases and Incidence Estimates for 2008 for Patients Aged 10 to 79 Years by Sex and Five Year Age Groups

	Cases	Person-Time (in 100,000 years)	Incidence (per 100,000 person-years)	95% Confidence Interval
Female	4818	24.64	195.57	[190.09; 201.17]
10 - 14 years	40	1.77	22.66	[16.19; 30.86]
15 - 19 years	375	1.63	229.92	[207.23; 254.41]
20 - 24 years	991	1.62	610.41	[572.99; 649.63]
25 - 29 years	906	1.88	481.57	[450.72; 513.98]
30 - 34 years	577	1.67	344.48	[316.94; 373.77]
35 - 39 years	493	2.30	214.59	[196.06; 234.39]
40 - 44 years	587	3.15	186.51	[171.72; 202.23]
45 - 49 years	348	2.73	127.63	[114.57; 141.76]
50 - 54 years	219	2.06	106.09	[92.51; 121.12]
55 - 59 years	120	1.70	70.54	[58.49; 84.35]
60 - 64 years	77	1.32	58.39	[46.08; 72.97]
65 - 69 years	57	1.50	38.07	[28.84; 49.33]
70 - 74 years	24	0.86	27.89	[17.87; 41.50]
75 - 79 years	4	0.45	8.95	[2.44; 22.92]

------------------------------------ continued on the next page ------------------------------------

	Cases	Person-Time (in 100,000 years)	Incidence (per 100,000 person-years)	95% Confidence Interval
---------- continued ----------				
Male	3826	26.57	144.00	[139.48; 148.64]
10 - 14 years	19	1.85	10.29	[6.19; 16.06]
15 - 19 years	130	1.73	75.36	[62.96; 89.48]
20 - 24 years	547	1.61	340.56	[312.62; 370.34]
25 - 29 years	792	1.78	446.01	[415.48; 478.18]
30 - 34 years	474	1.51	314.20	[286.55; 343.80]
35 - 39 years	486	2.02	241.11	[220.15; 263.53]
40 - 44 years	450	3.08	146.18	[132.98; 160.33]
45 - 49 years	329	2.89	113.87	[101.90; 126.86]
50 - 54 years	205	2.35	87.34	[75.79; 100.15]
55 - 59 years	156	2.05	76.09	[64.62; 89.01]
60 - 64 years	77	1.71	44.91	[35.45; 56.13]
65 - 69 years	107	2.08	51.50	[42.20; 62.23]
70 - 74 years	38	1.30	29.20	[20.67; 40.09]
75 - 79 years	16	0.63	25.31	[14.47; 41.10]

---------- continued on the next page ----------

	Cases	Person-Time (in 100,000 years)	Incidence (per 100,000 person-years)	95% Confidence Interval
--- continued ---				
Total	8644	51.20	168.81	[165.27; 172.41]
10 - 14 years	59	3.61	16.33	[12.43; 21.07]
15 - 19 years	505	3.36	150.47	[137.63; 164.18]
20 - 24 years	1538	3.23	476.21	[452.71; 500.62]
25 - 29 years	1698	3.66	464.30	[442.48; 486.93]
30 - 34 years	1051	3.18	330.13	[310.47; 350.71]
35 - 39 years	979	4.31	226.98	[212.99; 241.66]
40 - 44 years	1037	6.23	166.57	[156.58; 177.02]
45 - 49 years	677	5.62	120.55	[111.64; 129.98]
50 - 54 years	424	4.41	96.12	[87.18; 105.72]
55 - 59 years	276	3.75	73.58	[65.15; 82.79]
60 - 64 years	154	3.03	50.77	[43.07; 59.45]
65 - 69 years	164	3.57	45.88	[39.12; 53.46]
70 - 74 years	62	2.16	28.68	[21.99; 36.77]
75 - 79 years	20	1.08	18.54	[11.32; 28.63]

Table 5: Person-Time, Cases and Incidence Estimates for 2009 for Patients Aged 10 to 79 Years by Sex and Five Year Age Groups

	Cases	Person-Time (in 100,000 years)	Incidence (per 100,000 person-years)	95% Confidence Interval
Female	4285	24.44	175.35	[170.14; 180.68]
10 - 14 years	24	1.76	13.61	[8.72; 20.25]
15 - 19 years	222	1.45	152.84	[133.39; 174.32]
20 - 24 years	755	1.48	511.51	[475.67; 549.33]
25 - 29 years	909	1.94	467.92	[437.99; 499.35]
30 - 34 years	582	1.72	338.81	[311.84; 367.49]
35 - 39 years	444	2.06	215.95	[196.33; 237.01]
40 - 44 years	546	3.03	180.42	[165.60; 196.21]
45 - 49 years	360	2.85	126.30	[113.59; 140.05]
50 - 54 years	174	2.17	80.22	[68.75; 93.07]
55 - 59 years	100	1.74	57.57	[46.84; 70.02]
60 - 64 years	68	1.24	54.63	[42.43; 69.26]
65 - 69 years	63	1.54	41.04	[31.54; 52.51]
70 - 74 years	26	1.01	25.85	[16.88; 37.87]
75 - 79 years	12	0.46	26.09	[13.48; 45.58]

------------------------------------ continued on the next page ------------------------------------

	Cases	Person-Time (in 100,000 years)	Incidence (per 100,000 person-years)	95% Confidence Interval

--- continued ---

Male	3605	26.47	136.21	[131.80; 140.73]
10 - 14 years	20	1.84	10.84	[6.62; 16.75]
15 - 19 years	83	1.52	54.53	[43.43; 67.60]
20 - 24 years	446	1.47	303.09	[275.61; 332.56]
25 - 29 years	796	1.83	435.59	[405.86; 466.94]
30 - 34 years	532	1.59	334.03	[306.25; 363.66]
35 - 39 years	421	1.82	231.02	[209.48; 254.18]
40 - 44 years	448	2.95	152.04	[138.28; 166.79]
45 - 49 years	330	3.01	109.53	[98.03; 122.01]
50 - 54 years	188	2.43	77.25	[66.60; 89.12]
55 - 59 years	129	2.09	61.58	[51.41; 73.17]
60 - 64 years	79	1.68	47.13	[37.31; 58.74]
65 - 69 years	79	2.07	38.10	[30.17; 47.49]
70 - 74 years	36	1.49	24.19	[16.94; 33.49]
75 - 79 years	18	0.66	27.27	[16.16; 43.10]

------------------------------------ continued on the next page ------------------------------------

	Cases	Person-Time (in 100,000 years)	Incidence (per 100,000 person-years)	95% Confidence Interval
------ continued ------				
Total	7890	50.90	155.00	[151.60; 158.46]
10 - 14 years	44	3.61	12.20	[8.86; 16.37]
15 - 19 years	305	2.97	102.53	[91.35; 114.71]
20 - 24 years	1201	2.95	407.46	[384.73; 431.17]
25 - 29 years	1705	3.77	452.25	[431.04; 474.24]
30 - 34 years	1114	3.31	336.51	[317.04; 356.87]
35 - 39 years	865	3.88	223.03	[208.42; 238.41]
40 - 44 years	994	5.97	166.42	[156.23; 177.09]
45 - 49 years	690	5.86	117.69	[109.07; 126.80]
50 - 54 years	362	4.60	78.65	[70.76; 87.19]
55 - 59 years	229	3.83	59.76	[52.27; 68.02]
60 - 64 years	147	2.92	50.33	[42.52; 59.15]
65 - 69 years	142	3.61	39.35	[33.15; 46.38]
70 - 74 years	62	2.49	24.86	[19.06; 31.87]
75 - 79 years	30	1.12	26.79	[18.07; 38.24]

Table 6: Person-Time, Cases and Incidence Estimates for 2010 for Patients Aged 10 to 79 Years by Sex and Five Year Age Groups

	Cases	Person-Time (in 100,000 years)	Incidence (per 100,000 person-years)	95% Confidence Interval
Female	4842	26.76	180.95	[175.88; 186.12]
10 - 14 years	34	1.89	17.96	[12.44; 25.10]
15 - 19 years	200	1.34	148.95	[129.02; 171.09]
20 - 24 years	769	1.56	493.42	[459.16; 529.56]
25 - 29 years	1099	2.34	468.90	[441.59; 497.47]
30 - 34 years	716	2.17	329.54	[305.84; 354.59]
35 - 39 years	493	2.18	225.65	[206.17; 246.47]
40 - 44 years	547	3.19	171.41	[157.35; 186.40]
45 - 49 years	480	3.24	148.01	[135.07; 161.87]
50 - 54 years	235	2.48	94.67	[82.95; 107.58]
55 - 59 years	113	1.91	59.09	[48.70; 71.05]
60 - 64 years	64	1.26	50.69	[39.04; 64.73]
65 - 69 years	55	1.50	36.56	[27.54; 47.58]
70 - 74 years	28	1.16	24.21	[16.08; 34.98]
75 - 79 years	9	0.51	17.58	[8.04; 33.38]

------------------------------------ continued on the next page ------------------------------------

	Cases	Person-Time (in 100,000 years)	Incidence (per 100,000 person-years)	95% Confidence Interval
------------ continued ------------				
Male	4510	29.43	153.26	[148.82; 157.80]
10 - 14 years	19	1.96	9.68	[5.83; 15.12]
15 - 19 years	85	1.41	60.48	[48.31; 74.78]
20 - 24 years	504	1.53	330.02	[301.83; 360.13]
25 - 29 years	976	2.22	439.14	[412.02; 467.58]
30 - 34 years	802	2.19	365.54	[340.68; 391.74]
35 - 39 years	564	2.15	262.35	[241.14; 284.92]
40 - 44 years	529	3.24	163.22	[149.60; 177.74]
45 - 49 years	396	3.46	114.44	[103.44; 126.28]
50 - 54 years	238	2.76	86.23	[75.62; 97.91]
55 - 59 years	138	2.27	60.77	[51.06; 71.80]
60 - 64 years	95	1.75	54.38	[44.00; 66.48]
65 - 69 years	95	2.06	46.02	[37.23; 56.26]
70 - 74 years	53	1.67	31.75	[23.78; 41.53]
75 - 79 years	16	0.75	21.26	[12.15; 34.53]

------------ continued on the next page ------------

	Cases	Person-Time (in 100,000 years)	Incidence (per 100,000 person-years)	95% Confidence Interval
---------- continued ----------				
Total	9352	56.19	166.45	[163.09; 169.86]
10 - 14 years	53	3.85	13.75	[10.30; 17.98]
15 - 19 years	285	2.75	103.71	[92.01; 116.47]
20 - 24 years	1273	3.09	412.55	[390.19; 435.85]
25 - 29 years	2075	4.57	454.42	[435.07; 474.40]
30 - 34 years	1518	4.37	347.63	[330.36; 365.57]
35 - 39 years	1057	4.33	243.85	[229.37; 259.00]
40 - 44 years	1076	6.43	167.28	[157.44; 177.59]
45 - 49 years	876	6.70	130.68	[122.17; 139.63]
50 - 54 years	473	5.24	90.23	[82.28; 98.74]
55 - 59 years	251	4.18	60.01	[52.81; 67.91]
60 - 64 years	159	3.01	52.83	[44.94; 61.71]
65 - 69 years	150	3.57	42.03	[35.58; 49.32]
70 - 74 years	81	2.83	28.66	[22.76; 35.62]
75 - 79 years	25	1.26	19.77	[12.80; 29.19]
